# Regional Nerve Block for Hip Fracture Surgery in an Elderly Patient With Severe Aortic Stenosis: A Case Report

**DOI:** 10.7759/cureus.91187

**Published:** 2025-08-28

**Authors:** Ana Milosavljevic, Milos Blagojevic, Andrija Milicevic, Teodora Antic, Andreja Baljozovic

**Affiliations:** 1 Anesthesia and Critical Care, Institute for Orthopedic Surgery "Banjica", Belgrade, SRB; 2 Orthopedics and Traumatology, Institute for Orthopedic Surgery "Banjica", Belgrade, SRB; 3 Surgery, Faculty of Medicine, University of Belgrade, Belgrade, SRB

**Keywords:** aortic stenosis, elderly, hip fracture, peripheral nerve block, regional anesthesia

## Abstract

Proximal femoral fractures are most common in elderly patients and are associated with high mortality. Anesthesia strategy in the elderly focuses on minimising its effects on the already impaired cardiopulmonary reserve and cognitive function. We present the anesthesia management of an elderly patient with severe aortic stenosis and demonstrate the benefits of peripheral nerve block (PNB) in trochanteric fracture surgery.

A 90-year-old patient was admitted for surgery due to a trochanteric fracture. The anesthesia plan involved administering a PNB. The patient had a medical history of severe aortic stenosis with an aortic valve area of 0.84 cm², hypertension, and chronic kidney disease, which was complicated by melena after admission. After gastroenterological treatment and resolution of symptoms, the patient was scheduled for orthopedic surgery. An ultrasound-guided PNB was performed, which included the fascia iliaca block, femoral nerve block, pericapsular nerve group block, and lumbar plexus block. A total of 35 mL of levobupivacaine at a concentration of 0.25% was applied for the PNB, after which complete anesthesia of the left hip and thigh region was achieved. Six hours after surgery, the pain score measured by a numeric rating scale was 3; analgesia was maintained with paracetamol and a reduced dose of metamizole intravenously. PNB achieves adequate conditions for trochanteric fracture surgery and postoperative analgesia, while preserving hemodynamic stability, which enables early rehabilitation and better recovery after surgery.

## Introduction

Proximal femoral fractures account for half of all hip fractures. They are most common in elderly patients as one of the typical fragility fractures. These injuries are associated with high mortality - about 30% at one year - due to physiological and pathological changes associated with old age [1].

Anesthesia poses significant challenges for the elderly. Adequate preoperative evaluation is required and should be a multidisciplinary approach involving an anesthesiologist, an orthopedic surgeon, and a specialist in internal medicine. An anesthesia strategy in the elderly involves providing sufficient conditions for surgery while minimising its effects on the already impaired cardiopulmonary reserve and cognitive function [2,3].

The choice of anesthesia for hip fracture fixation is often regional - most commonly spinal anesthesia or peripheral nerve block (PNB). Spinal anesthesia leads to sympathicolysis, causing a decrease in vascular resistance and thereby reducing perfusion pressure. This is particularly significant in elderly patients, especially those with valvular heart disease. Therefore, the use of spinal anesthesia in patients with severe aortic stenosis is considered a relative contraindication [4,5].

PNB has a less significant impact on vital organs, making it safer for patients with existing comorbidities and reducing systemic analgesic requirements. This method is rarely used as the sole anesthesia technique; it is usually combined with general anesthesia to provide better postoperative analgesia and avoid the use of opioids. PNB for hip fracture surgery includes the femoral nerve block (FNB), fascia iliaca block (FIB), lumbar plexus block (LPB), and a newer method - the pericapsular nerve group (PENG) block [6,7].

In this paper, we present the anesthesia management of an elderly patient with severe aortic stenosis who underwent trochanteric fracture surgery. The primary goal was to maintain hemodynamic stability. Additionally, we aimed to demonstrate the benefits of PNB in this patient population.

## Case presentation

**Figure 1:**
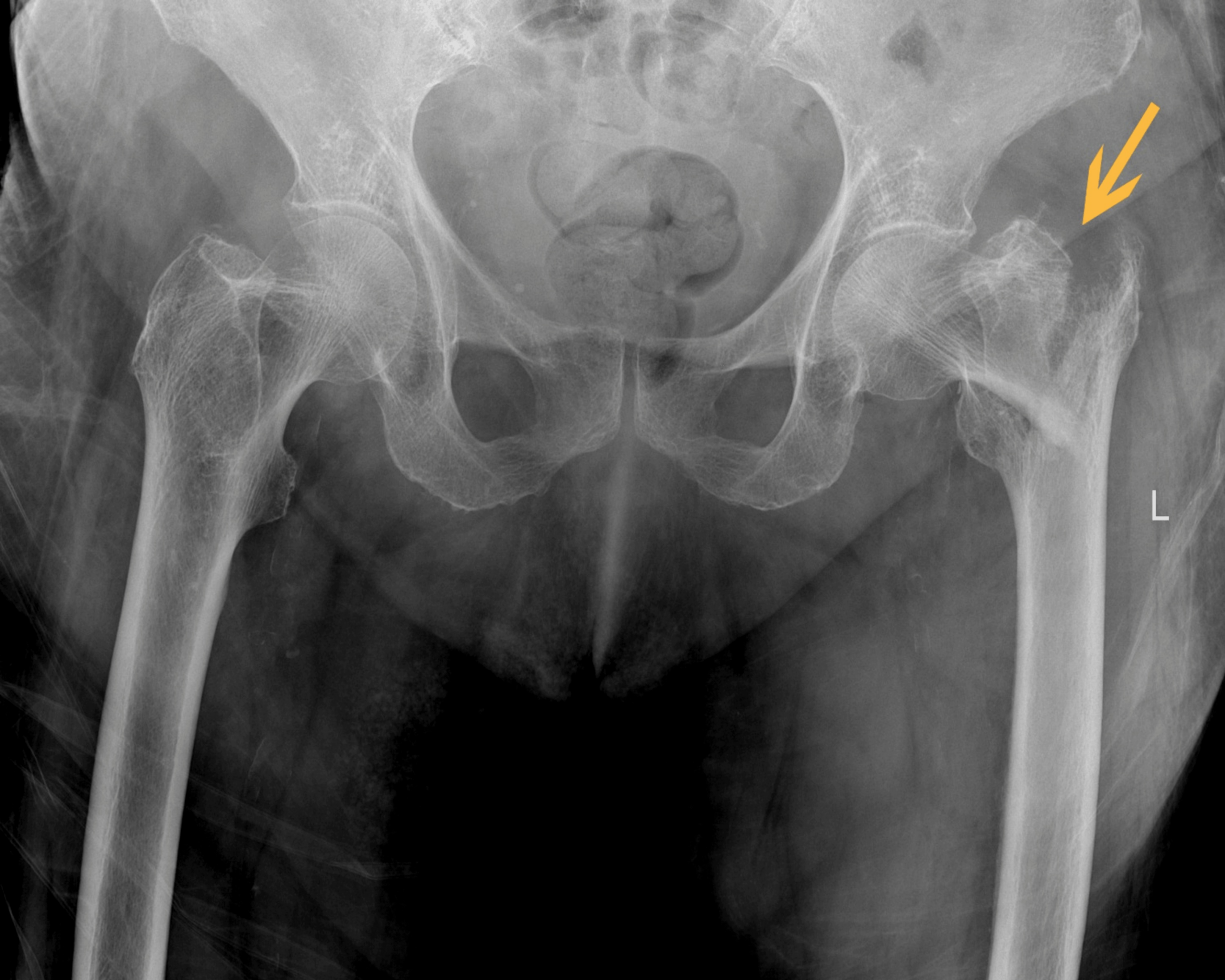
A pelvic radiography A yellow arrow showing an unstable trochanteric fracture of the left hip.

A 90-year-old female patient with a body mass index of 25.22 kg/m² (weight: 67 kg; height: 163 cm) was admitted to the Emergency Department due to pain and limited mobility in the left hip area, as well as leg shortening, following a fall on a flat surface earlier that day. Pelvic radiography showed an unstable trochanteric fracture of the left hip (Figure 1).

**Figure 2:**
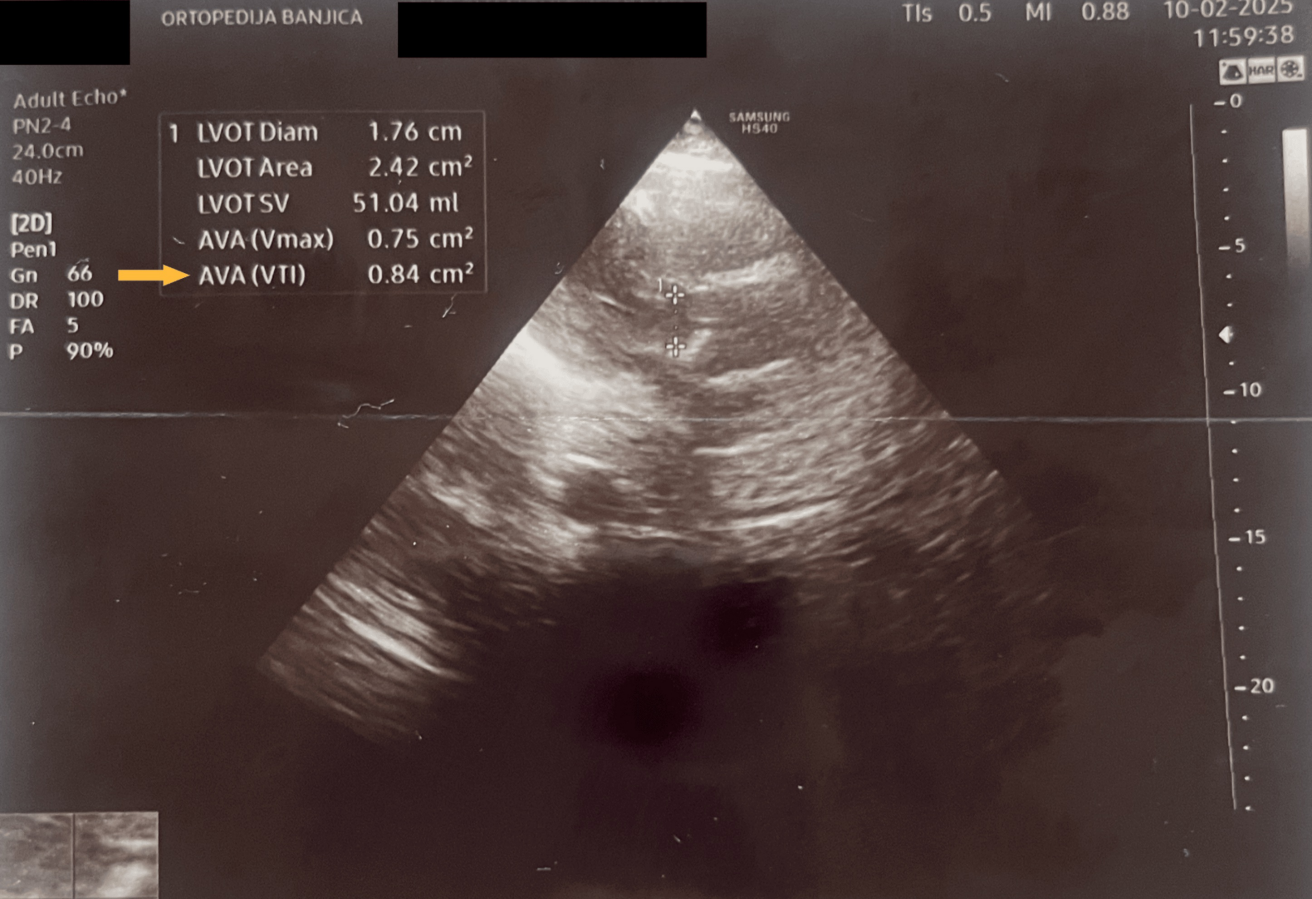
Echocardiographic image showing the aortic valve On echocardiography, aortic stenosis was confirmed; the yellow arrow indicates the measured aortic valve area (AVA), which is 0.84 cm^2^.

**Figure 3:**
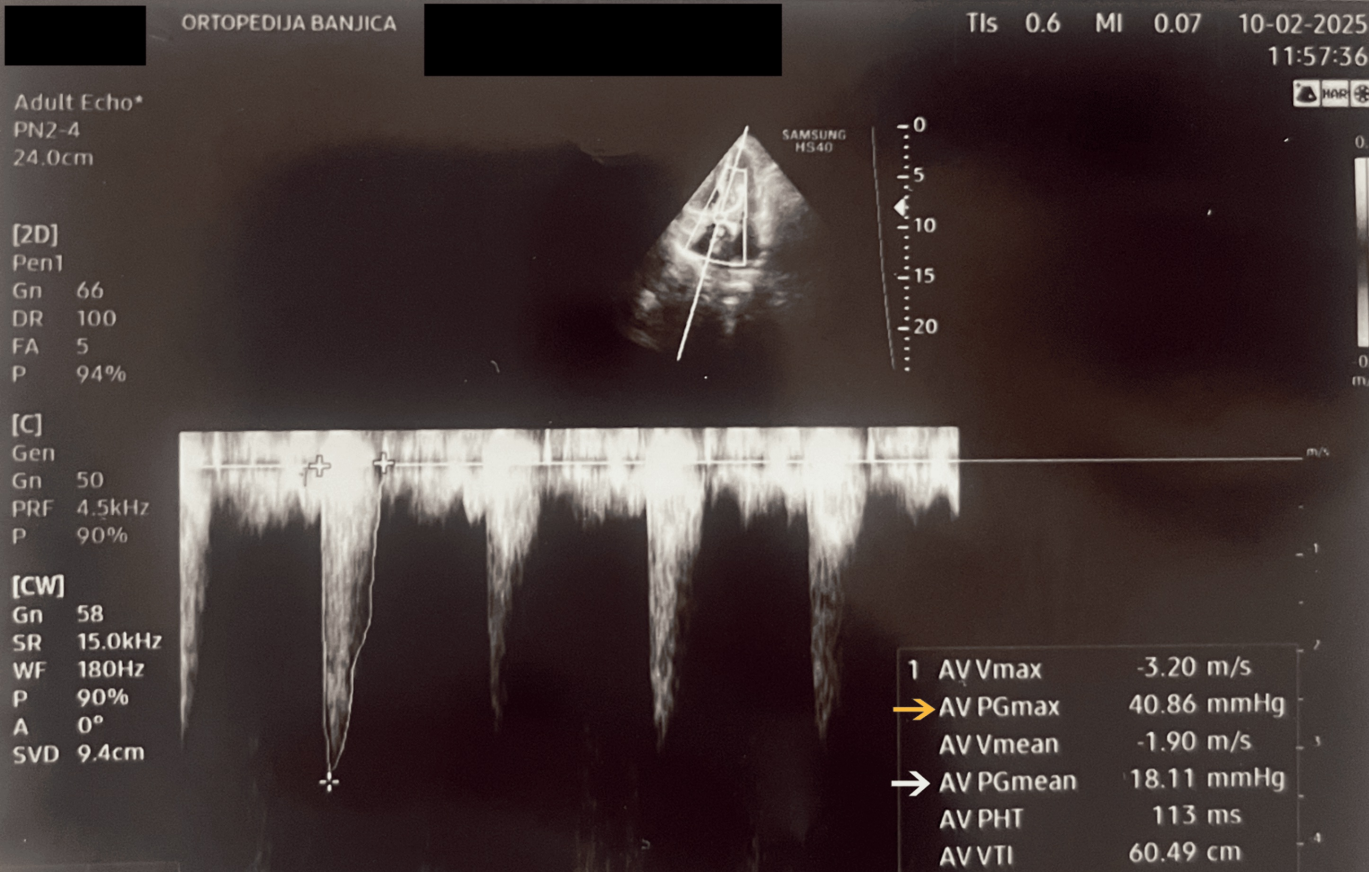
Echocardiographic image of the pressure gradient through the aortic valve In the image, the yellow arrow indicates the maximum pressure gradient through the aortic valve (40.86 mmHg), while the white arrow represents the mean pressure gradient (18.11 mmHg).

The surgical plan included closed reduction and internal fixation (CRIF) with a cephalomedullary nail. Thromboprophylaxis with low-molecular-weight heparin (LMWH) - nadroparin calcium 2850 U/0.3 mL by subcutaneous injection every 12 hours - and proton pump inhibitors (pantoprazole 40 mg by intravenous injection every 12 hours) was initiated. On admission, the patient was conscious and hemodynamically and respiratorily stable. She had a medical history of severe aortic stenosis, hypertension, and chronic kidney disease. Serum urea concentration was 13.4 mmol/L, and creatinine was 136 µmol/L. Her blood pressure on admission was 140/90 mmHg. Transthoracic echocardiography showed an aortic valve area of 0.84 cm² (Figure 2), a mean pressure gradient of 19 mmHg, and a maximum pressure gradient of 40 mmHg (Figure 3). Patient characteristics and clinical data are summarised in Table 1.

**Table 1:** Patient characteristics and clinical data ASA: American Society of Anesthesiologists; NRS: numeric rating scale; FIB: fascia iliaca block; FNB: femoral nerve block; LPB: lumbar plexus block; PENG: pericapsular nerve group

Parameter	Data
Age	90
Sex	Female
ASA classifications	3
NRS on admission to the ICU	0
NRS 18 hours after surgery	3
Dosing for FIB	0.25% levobupivacaine 10 mL
Dosing for FNB	0.25% levobupivacaine 5 mL
Dosing for LPB	0.25% levobupivacaine 10 mL
Dosing for PENG block	0.25% levobupivacaine 10 mL
Block coverage	L1-S3
Type of surgery	Closed reduction and fixation with a cephalomedullary nail
Surgery duration (min)	35
Anesthesia type	Regional anesthesia

After admission on day 3, the condition became further complicated by melena. She was referred for a gastroenterology examination. Gastroscopy revealed a stomach ulcer, for which treatment with proton pump inhibitors was prescribed. After the gastroenterological treatment and resolution of symptoms, the patient was scheduled for orthopedic surgery on the seventh day after admission. She was classified as an American Society of Anesthesiologists (ASA) 3, based on her medical history and physical status. Premedication with a sedative was not prescribed. Two intravenous cannulas and a urinary catheter were placed. Tranexamic acid, 500 mg intravenously, was prescribed over 30 minutes. Standard noninvasive monitoring was conducted (noninvasive blood pressure, pulse oximetry, and continuous electrocardiogram). Crystalloid infusions and antibiotics (first-generation cephalosporin - cefazolin, 50 mg/kg/day intravenously) were started before surgery.

**Figure 4:**
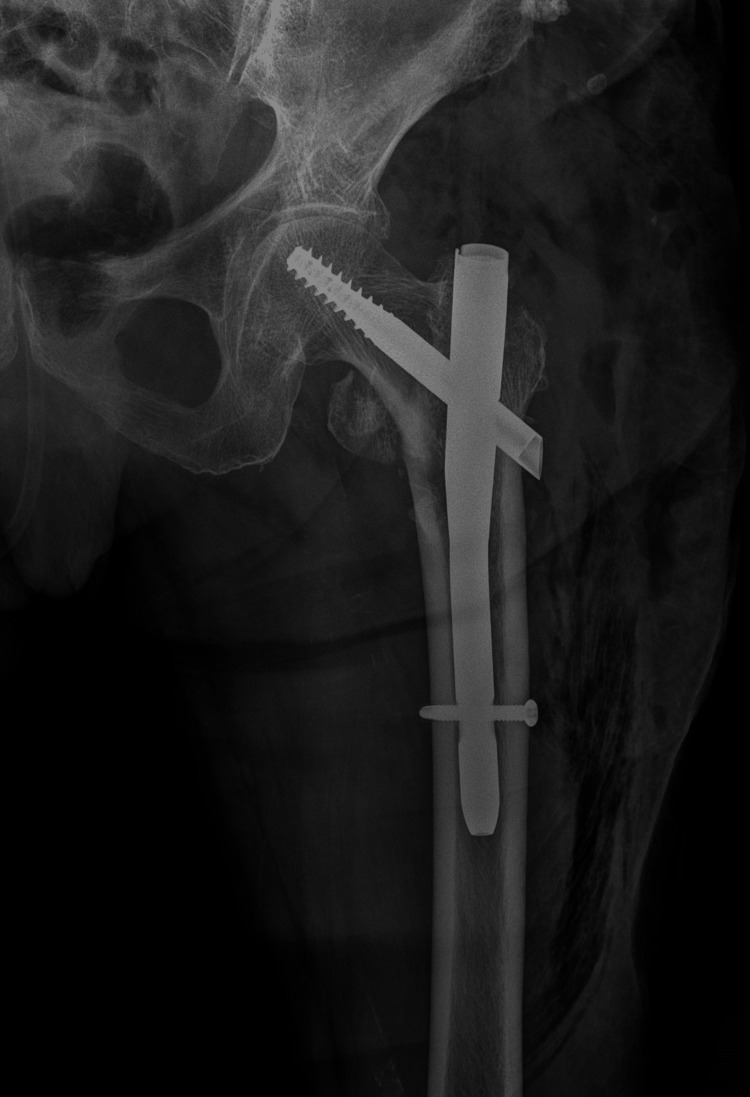
Postoperative radiograph of the left hip showing fracture fixation with a cephalomedullary nail

First, an ultrasound-guided FIB (eZono®4000; eZono AG, Jena, Germany) was performed in the supine position through an infra-inguinal approach. The probe was placed over the inguinal ligament. The probe was moved distally until the iliac fascia and the iliopsoas muscle were identified. The needle was advanced to below the iliac fascia while injecting the local anesthetic levobupivacaine 0.25%, 10 mL. Then, an ultrasound-guided FNB was performed in the supine position. After the identification of the femoral vein and artery, the probe was moved laterally to identify the femoral nerve. The needle was inserted at an approximately 30-degree angle, and levobupivacaine 0.25%, 10 mL was injected perineurally. At last, the probe was transversal over the anterior superior iliac spine. In the space between the iliopsoas tendon anteriorly and the pubic ramus posteriorly, local anesthetic levobupivacaine 0.25%, 5 mL was injected for the PENG block. Five minutes after PNB, the patient denied feeling pain in the area of the femur fracture. Then, the patient was placed in the lateral decubitus position. A posterior LPB was performed using anatomical landmarks and peripheral nerve stimulation (Stimuplex® HNS 12; B. Braun, Bethlehem, PA, USA). Anatomic landmarks for determining the point of needle insertion are the iliac crest and the L3-L4 spinous processes. The nerve stimulator was set at 1.5 mA. A 22G, 10 cm needle (Stimuplex® Ultra 360® 22 Ga; B. Braun, Bethlehem, PA, USA) was used. The needle insertion point was 3.5-4 cm lateral to the interspace processes. The needle was inserted at a perpendicular angle to the skin. The needle was advanced while quadriceps contractions were obtained at a depth of about 6 cm. At this point, after negative aspiration, local anesthetic levobupivacaine 0.25%, 10 mL was injected perineurally. A total of 35 mL of anesthetic was applied for the PNB, after which complete anesthesia of the left hip and thigh region was achieved. During the operation, the patient was breathing spontaneously. Oxygen at 6 L/min was administered through the face mask, and intravascular volume was maintained with crystalloid solutions (Ringer's lactate and sodium chloride 0.9%). Hemodynamic stability was achieved intraoperatively, with a mean arterial pressure of 75-80 mmHg, a heart rate of approximately 80 beats per minute, and an SpO_2_ of 99%. CRIF with a cephalomedullary nail was performed (Figure 4).

The operation lasted 35 minutes. Postoperatively, the patient was observed in the ICU for six hours. The pain score was measured using a numeric rating scale (NRS), which was 0 on admission to the ICU. In the ward, the NRS was 3 at 12 hours after surgery, and the postoperative pain control strategy included paracetamol (1 g intravenously every eight hours) and metamizole (2.5 g intravenously every 24 hours). Metamizole was given with caution, in a reduced dose, once a day for a total of two days. Other intravenous non-steroidal anti-inflammatory drugs (NSAIDs) were not administered because of the risk of recurrent gastrointestinal bleeding. Early patient ambulation with partial weight-bearing began on the second postoperative day. On the fifth postoperative day, after a satisfactory clinical and laboratory check-up, the patient was discharged home. Thromboembolic prophylaxis was continued until the 21st day.

## Discussion

Trochanteric hip fractures in the elderly are associated with increased morbidity and mortality as a result of factors such as patient age, trauma, surgical intervention, comorbidities, and reduced functional reserve [1]. It is essential to adequately treat this type of fracture - if possible - within the first 48 hours from injury, because a delay in operation is associated with an increased risk of mortality [8]. The modern standard in treating a trochanteric fracture is CRIF with a cephalomedullary nail. This surgical technique is minimally invasive, avoiding exposure of the fracture site, which results in less tissue damage, earlier postoperative mobilisation, and reduced morbidity [7,9].

The primary goal for the anesthesiologist in patients with aortic stenosis is to maintain hemodynamic stability. Hypovolemia and hypotension should be avoided, and if they occur, they should be treated urgently due to the relatively fixed flow across the stenotic valve. Tachycardia and the sympathetic response to laryngoscopy, pain, and surgical stress must be prevented, as these conditions can decrease diastolic filling time [10]. By choosing regional anesthesia, the sympathetic response to surgical stress can be reduced, and intraoperative and postoperative pain control is improved. However, there is a risk of hypotension. Reduced cardiovascular compensatory mechanisms in the elderly significantly increase the degree of hypotension caused by sympathetic block in spinal anesthesia. Better hemodynamic control is achieved with general anesthesia. After surgery under general anesthesia, somnolence, nausea, vomiting, and agitation may occur, and there is a risk of postoperative cognitive dysfunction and delirium [11]. PNB was found to have a lesser influence on hemodynamics, respiratory function, and consciousness. The choice of anesthesia must be individualised for each patient [10,11].

Regional anesthesia, which includes neuraxial anesthesia (spinal anesthesia and epidural anesthesia) and PNBs, provides adequate conditions for trochanteric fracture surgery. Both types of regional anesthesia may lead to a reduction in systemic analgesic requirements and opioid-related side effects. PNB has a less significant impact on vital organs, making it safer for patients with existing comorbidities [12]. Since our patient had an unstable trochanteric fracture and severe aortic stenosis, our choice of anesthesia was a PNB involving the FNB, FIB, LPB, and the PENG block. Ultrasound-guided regional anesthesia increases the chance of success of PNBs in general. The FIB provides adequate perioperative and postoperative analgesia. A local anesthetic is applied to the potential space between the iliacus muscle and the fascia iliaca, within which the femoral nerve and lateral femoral cutaneous nerve are located [7,13]. An LPB can provide anesthesia or analgesia to the anterolateral and medial aspects of the thigh, which achieves sufficient muscle relaxation and adequate analgesia for hip surgery [14]. The FNB provides anesthesia to the anterior portion of the upper leg. It is easy to perform, but the most crucial disadvantage is that it causes quadriceps weakness. It is used in combination with other blocks to achieve adequate anesthesia. Today, preference is given to the PENG block, which targets the articular branches of the femoral nerve, obturator nerve, and accessory nerve, which pass over the anterior capsule of the hip joint [15]. In their study, Tavares et al. [16] evaluated pain in patients with trochanteric fractures, comparing a group without a PENG block with a group that received a PENG block, and concluded that the use of a PENG block reduced postoperative pain and enabled earlier mobilisation. Although trochanteric fractures are classified as extracapsular fractures, studies have shown that the PENG block achieves effective postoperative pain control without affecting motor function [6,15-17].

Postoperative pain management was complex. Because of gastrointestinal bleeding and renal impairment, we used metamizole with caution and in a reduced dose. Also, the goal was to avoid the use of opioids due to their side effects, such as nausea, vomiting, urinary retention, and respiratory depression. The application of regional analgesia reduces the need for systemic administration of drugs. PNB achieves adequate analgesia while preserving motor strength and hemodynamic stability, which enables early rehabilitation and better recovery after surgery [18]. Levobupivacaine is a good choice for patients with renal impairment, as it is metabolised in the liver to inactive metabolites and then excreted in the urine [19]. Bupivacaine was not the local anesthetic of choice because of its cardiotoxicity and neurotoxicity [20]. As sensitivities to local anesthetics are increased in the elderly due to a decrease in nerve conduction velocity, a reduced concentration of levobupivacaine was sufficient to achieve an adequate block [19,20]. Because of all the above, we used diluted levobupivacaine (concentration 0.25%). The reason is the greater sensitivity of the elderly to local anesthetics. The local anesthetic of choice is levobupivacaine due to its lower cardiotoxicity compared to bupivacaine.

Due to these facts, we tried to avoid general anesthesia because of postoperative discomfort and the need for analgesics. Regional anesthesia provides good pain control; however, due to severe aortic stenosis, we decided not to use spinal anesthesia. In order to achieve adequate anesthesia and analgesia for surgery, as well as good postoperative pain control - without the use of opioid analgesics and NSAIDs - we decided to apply four PNB techniques.

As we already mentioned, we measured pain using the NRS. Since the highest reported pain score was 3, we conclude that the patient experienced only mild pain. A limitation of this article is that it does not consider the application of PNB without an ultrasound device.

## Conclusions

The anesthesiologist's challenge was to adapt the anesthesia to the patient's health condition and to determine which anesthetic technique would have the least impact on the cardiovascular, renal, and gastrointestinal systems, while still being adequate for surgery. For trochanteric fracture surgery, effective anesthesia was accomplished through PNB, utilising techniques such as FNB, FIB, LPB, and the PENG block. These methods ensured good hemodynamic stability, which is crucial for patients with severe aortic stenosis. Adequate analgesia was achieved by nerve blockade; we used paracetamol and metamizole with caution, as these medications carry risks of kidney damage and gastrointestinal bleeding. In our case, this anesthesia technique not only achieved the most important goals but also contributed to reduced postoperative pain, earlier mobilisation, and improved functional recovery.
